# Prediction of Femoral Strength Based on Bone Density and Biochemical Markers in Elderly Men With Type 2 Diabetes Mellitus

**DOI:** 10.3389/fbioe.2022.855364

**Published:** 2022-03-28

**Authors:** Shaowei Jia, He Gong, Yingying Zhang, Hongmei Liu, Haipeng Cen, Rui Zhang, Yubo Fan

**Affiliations:** ^1^ Key Laboratory for Biomechanics and Mechanobiology of Ministry of Education, Beijing Advanced Innovation Center for Biomedical Engineering, School of Biological Science and Medical Engineering, Beihang University, Beijing, China; ^2^ Beijing Key Laboratory of Rehabilitation Technical Aids for Old-Age Disability, National Research Center for Rehabilitation Technical Aids, Beijing, China; ^3^ Rehabilitation Hospital, National Research Center for Rehabilitation Technical Aids, Beijing, China

**Keywords:** type 2 diabetes mellitus, quantitative computed tomography, finite element analysis, femoral strength, bone turnover

## Abstract

**Purpose:** Effects of bone density, bone turnover and advanced glycation end products (AGEs) on femoral strength (FS) are still unclear in patients with type 2 diabetes mellitus (T2DM). This study aims to assess and predict femoral strength and its influencing factors in elderly men with T2DM.

**Methods:** T2DM patients (*n* = 10, mean age, 66.98 years) and age-matched controls (*n* = 8, mean age, 60.38 years) were recruited. Femoral bone mineral density (BMD) and serum biochemical indices of all subjects were measured. FS was evaluated through finite element analysis based on quantitative computed tomography. Multiple linear regression was performed to obtain the best predictive models of FS and to analyze the ability of predictors of FS in both groups.

**Results:** FS (*p* = 0.034), HbA1c (*p* = 0.000) and fasting blood glucose (*p* = 0.000) levels of T2DM group were significantly higher than those of control group; however, the P1NP level (*p* = 0.034) was significantly lower. FS was positively correlated with femoral neck T score (FNTS) (r = 0.794, *p* < 0.01; r = 0.881, *p* < 0.01) in both groups. FS was correlated with age (r = -0.750, *p* < 0.05) and pentosidine (r = -0.673, *p* < 0.05) in T2DM group. According to multiple linear regression, FNTS and P1NP both contributed to FS in two groups. P1NP significantly improved the prediction of FS in both groups, but significant effect of FNTS on predicting FS was only presented in control group. Furthermore, pentosidine, age and HbA1c all played significant roles in predicting FS of T2DM.

**Conclusion:** Femoral strength was higher in elderly men with T2DM, which might be caused by higher BMD and lower bone turnover rate. Moreover, besides BMD and bone formation level, AGEs, blood glucose and age might significantly impact the prediction of femoral strength in T2DM.

## Introduction

Diabetes mellitus (DM) is one of the most common metabolism diseases. Type 2 diabetes mellitus (T2DM) is complicated and universal, so it has been considered as a health concern (Glovaci et al., 2019). Furthermore, T2DM is often accompanied with high bone fracture risk. Accurate prediction of bone strength could provide important information for preventing bone fracture in T2DM. Previous investigations proved that T2DM had complicated effects on bone because of many factors, including bone metabolism, insulin level, and advanced glycosylation end products (AGEs) (Li et al., 2013). Sufficient understanding of bone strength of T2DM and its influencing factors is necessary for predicting bone strength in clinics.

Strength is one of the most important indices of bone mechanical properties. It reflects the ability of bone to resist deformation and fracture. It could provide important basis for assessing bone quality and protecting bones in patients with T2DM to investigate the relationship between bone strength and its influencing factors. Bone strength was mainly affected by bone mineral density (BMD) and bone quality, and bone quality was determined by bone morphological parameters and material properties (Farr and Khosla, 2016). In clinics, the assessment of bone strength was based on BMD measured *via* clinical dual-energy X-ray (DXA). However, some limitations have been observed. DXA only displayed the 2D projection of 3D complex structures and failed to distinguish soft tissues, cancellous bones, and cortical bones (Kanis et al., 2011). Moreover, BMD measured *via* DXA was insensitive to short-term physical changes in bone strength through certain treatments (Bolotin, 2007). As a noninvasive method to evaluate 3D morphology, quantitative computed tomography (QCT) was widely used to analyze bone morphology and to assess spatial distribution of bone density (Ramamurthi et al., 2012). Moreover, finite element analysis (FEA) based on QCT images was a FDA-approved approach to noninvasively estimate changes in bone strength. QCT-based FEA has been successfully applied to assessing bone strength in previous studies (Keaveny et al., 2014; Knowles et al., 2021). Therefore, QCT-based FEA was reasonable and effective in examining bone strength with changed bone material properties in elderly men with T2DM.

Bone turnover continuously occurred to maintain bone dynamic balance, and it could replace damaged bone. This process consisted of bone formation and bone resorption. Many studies have indicated that the bone turnover rate in patients with T2DM was lower than that in individuals without T2DM (Shu et al., 2012; Farr et al., 2014; Wang et al., 2019). Histomorphometry has confirmed that low bone turnover and formation rates with reduced osteoclast activity were due to a low degree of matrix accumulation instead of abnormal bone mineralization (Krakauer et al., 1995). Therefore, it might be worth further verifying whether a low bone turnover rate could lead to reductions in bone quality and strength. In contrast, a previous study indicated that bone turnover markers (BTMs) and bone turnover rate did not differ between patients with T2DM and control subjects (Starup-Linde and Vestergaard, 2016). Thus, characteristics of bone turnover should be further clarified, and the ability of bone turnover on predicting bone strength in T2DM should also be investigated.

AGEs are composite products formed *via* the Maillard reaction after proteins are modified by aldose sugars *via* nonenzymatic chemical modification. AGEs are also considered as a factor of increasing bone fragility. A high AGEs level was found in patients with T2DM, moreover, a previous study indicated that high AGEs concentration could increase bone stiffness and bone fragility and reduce bone formation because of the suppression of osteoblast activity by AGEs (Ogawa et al., 2007). Pentosidine was a typical AGEs marker, it was significantly increased in non-diabetic patients with hip fracture compared with non-diabetic patients without fracture in the clinical setting, which indicated that increase in the non-enzymatic crosslinked type of AGEs might be a cause of deterioration of bone strength (Yamamoto and Sugimoto, 2016). Due to the difficulty of assessing bone strength *in vivo*, there was still no convinced explanation on the relationship between AGEs and bone strength. In consideration of the accuracy of assessing bone strength *via* QCT-based FEA, investigation of the effect and predictive ability of AGEs on simulated bone strength might be valuable for explaining the relationship between AGEs and bone strength.

In this study, subject-specific femoral strength was evaluated through FEA based on QCT images, and differences in body indices, serum biochemical markers, and femoral strength were compared between T2DM and control groups to investigate the influences of BMD, bone turnover, and serum biochemical markers on the femoral strength of the elderly men with T2DM. The relationships of femoral strength with BMD, bone turnover, and serum biochemical markers were analyzed. Moreover, the best predictive models of femoral strength of T2DM and control groups were obtained by multiple linear regression. In this study, the significant predictors of femoral strength could be found out, and it could provide sufficient evidence for assessing femoral strength of the elderly men with T2DM in clinics.

## Materials and Methods

### Subjects

This study was approved by the ethics committee of the affiliated hospital of the National Research Center for Rehabilitation Technical Aids and conformed to the Declaration of Helsinki. Patients with T2DM (*n* = 10) and control subjects without T2DM (*n* = 8) were recruited in this hospital. All the subjects signed informed consent form. The specific recruitment criteria of control and T2DM subjects were as follows: male subjects; age ≥50 years; no bone diseases and no metal implants in their bodies; and the subjects of T2DM with a duration of T2DM of more than 5 years. Subjects with any of the following conditions were excluded: 1) those taking bone-affecting drugs, including hormone therapy, calcitonin, selective estrogen receptor modulators, parathyroid hormone, and bisphosphonates in the past 1 year; 2) long-term treatment with systemic glucocorticoid (3 months, dose ≥2.5 mg/day); 3) metastatic tumor in the past 5 years; 4) Paget’s syndrome; 5) untreated malabsorption syndrome; 6) hyperparathyroidism or hypoparathyroidism; and 7) history of renal injury (Pritchard et al., 2012).

### DXA Assessment

The femoral BMD and femoral neck T score of the left femur of the subjects were obtained *via* a DXA scanner (Norland X800, Norland Inc. United States).

### Imaging the Proximal Femurs

**FIGURE 1:**
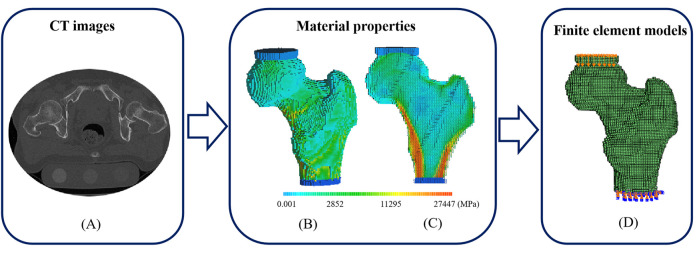
A typical CT image, distributions of elastic modulus and the corresponding FE model with loads and boundary conditions. **(A)** A transverse CT image of a proximal femur **(B)** Elastic modulus distribution of a proximal femur **(C)** Elastic modulus distribution of a proximal femur in cross-section **(D)** FE model with compressive deformation on the top surface of polymethylmethacrylate (PMMA) pad for femoral head and fixed bottom surface of PMMA pad for distal femur.

All images of the proximal femurs were collected using a clinical CT scanner (Optima CT680, GE medical system, Milwaukee, WI, United States). The scanning parameters were as follows: 140 kVp, intelligent scanning current (automatically selected on the basis of the thickness of different parts of the human body), 512 × 512 matrix, scanning thickness of 1.25 mm, and reconstructed slice thickness of 0.625 mm. During the scanning, a calibration phantom (QRM, Germany) containing calibration cells with 0.05, 0.1, and 0.2 g/cm^3^ equivalent concentrations of calcium hydroxyapatite was placed under the hip joint of the subjects. The calibration phantom and the subjects were scanned simultaneously. A typical transverse CT image was shown in Figure 1A.

### Biochemical Measurements

The fasting blood samples (before 9 a.m.) of all subjects were collected for measuring the following indices, such as blood routine indices including glycated hemoglobin (HbA1c), fasting plasma glucose (FPG), calcium (Ca), phosphorus (P), magnesium (Mg), C-peptide and Creatinine, BTMs including type I N-terminal propeptide (P1NP), β C-terminal cross-linked telopeptide of type I collagen (β-CTX), osteocalcin (OCN), bone alkaline phosphatase (BALP) and tartrate-resistant acid phosphatase 5b (TRAP5b), AGEs including total AGEs and pentosidine, and other regulating hormones of bone metabolism including intact parathyroid hormone (iPTH), 25-hydroxyvitamin D (25(OH)D), and sclerostin. Blood routine indices were determined *via* the photoelectric method, and BTMs and AGEs were measured using ELISA kits (Cusabio, Wuhan, China).

### Establishment of FE Models

Mimics 17.0 software (Materialise Inc. Leuven, Belgium) was used to establish 3D models of the left proximal femurs. Femoral models were meshed with 1.5 mm hexahedral elements. PMMA pads (E = 2,500 MPa, v = 0.3) having elements with the same size as those of the femurs were used to simulate the PMMA pads in mechanical tests (Crawford et al., 2003; Keaveny et al., 2014).

For femoral material assignment, apparent density (ρ_apparent_) was obtained from the relationship between the density of calibration phantom and the Hounsfiled Unit (HU) value, and ash density (ρ_ash_) was calculated using the following equation: ρ_ash_ = 1.22ρ_apparent_ + 0.0526 (Mirzaei et al., 2015). The elastic modulus (E) and yield strength (σ) of the femoral voxel were calculated on the basis of an empirical formula, as shown in Table 1. The femurs were set with 120 kinds of materials, and Poisson’s ratio was 0.4 (Gong et al., 2012). A typical distribution of femoral materials was shown in Figures 1B,C. After material assignment, the femoral models were imported into ABAQUS 6.14 (SIMULIA Inc. United States). In a compressive experiment, tie constraints were set between PMMA pads and femoral models, and all the degrees of freedom were fixed at the bottom surface of the PMMA pads for distal femurs, as shown in Figure 1D. Femoral strength was defined as the reaction force at a 4% compressive deformation (compressive displacement divided by femoral height) (Keaveny et al., 2014).

**TABLE 1:** Material properties of femur (Gong et al., 2012).

Material properties of femur				
ρ_ash_ (g/cm^3^)	ρ_ash_ = 0	0<ρ_ash_ ≤ 0.27	0.27<ρ_ash_ ≤ 0.6	0.6<ρ_ash_
Elastic modulus (MPa)	0.001	33900ρ^2.2^	5307ρ+469	10200ρ^2.01^
ρ_ash_ (g/cm^3^)	ρ_ash_ < 0.317	ρ_ash_ ≥ 0.317	—	—
Yield strength (MPa)	σ = 137ρ_ash_^1.88^	σ = 114ρ_ash_^1.72^	—	—

### Statistics

Nonparametric tests were used when the parameters were not met normal distribution, small size was mostly the main reason for nonnormal distribution of parameters (Ramachandran and Tsokos, 2015). Moreover, nonparametric tests were widely accepted and used to conduct the statistics analyses with small size samples (Pett, 1997). Therefore, the Mann-Whitney *U* test of nonparametric independent samples was conducted to compare the differences in the femoral BMD, femoral neck T score, femoral strength, and biochemical markers of T2DM group (*n* = 10) and control group (*n* = 8). The measured parameters were presented as quartiles. Moreover, nonparametric Spearman correlation analysis was carried out to analyze the correlations of all the parameters associated with femoral strength of both groups. Multiple linear regression analysis was used to obtain the best models of control and T2DM groups for predicting femoral strength and to analyze the main predictors of femoral strength.

## Results

### Differences Analysis

Differences in all the parameters of T2DM and control groups were listed in Table 2. Bone formation marker P1NP level, HbA1c level, fasting blood glucose (FBG) level, and femoral strength significantly differed between the two groups. The HbA1c level (*p* = 0.000), FBG level (*p* = 0.000), and femoral strength (*p* = 0.034) of T2DM group were significantly higher than those of control group. However, the P1NP level (*p* = 0.034) of T2DM group was significantly lower than that of control group.

**TABLE 2:** All parameters of subjects and the differences of parameters between T2DM and control groups.

Basic body parameters	T2DM group (n = 10)	Control group (n = 8)	*p* value
Age (years)	68 (53.75, 71.00)	62 (56.25, 63.75)	0.101
Height (cm)	172 (169, 175)	172 (166, 175)	0.868
Weight (kg)	75 (70, 82.75)	69 (62.75, 72.5)	0.055
BMI (kg/cm^2^)	25.69 (24.74, 26.9)	23.72 (21.25, 25.32)	0.068
Duration (year)	10 (7.25, 16.25)	—	—
Treatment	Metformin (*n* = 4); Insulin (*n* = 2); Non-treatment (*n* = 4)	—	—
Femoral BMD (mg/cm^2^)	1,016.5 (869.5,1149.0)	935 (885, 965.87)	0.829
Femoral neck T score	0.66 (-2.56, 0.038)	-1.79 (-2.10, -0.98)	0.616
Femoral strength (N)	8,945 (6,339.25, 10211.75)	6,867 (6,222.7,7497.5)	0.034*
**Biochemical indexes**			
HbA1c (%)	7.95 (7.22, 9.3)	5.6 (5.4, 5.9)	0.000*
FPG (mmol/L)	8.45 (7.1, 11.5)	4.99 (4.59, 5.1)	0.000*
P1NP (ng/ml)	33.28 (30.82, 35.06)	54.3 (34.8, 78.92)	0.034*
β-CTX (ng/ml)	0.11 (0.05, 0.12)	0.15 (0.07, 0.26)	0.408
OCN (ng/ml)	14.9 (11.42, 17.02)	18.3 (14.8, 23.35)	0.101
BALP (μg/L)	10.48 (8.96, 14.92)	7.51 (4.99, 13.38)	0.122
TRAP5b (U/I)	0.9 (0.83, 1.03)	1.44 (0.89, 2.2)	0.101
Total AGEs (ug/ml)	0.805 (0.34, 2.08)	0.56 (0.36, 1.56)	0.696
Pentosidine (pmol/ml)	574.5 (460.7, 634.9)	616.9 (412.1,841.7)	0.762
Sclerostin (pg/ml)	679.4 (491.5,813.7)	829.7 (662.2,1161.9)	0.408
iPTH (pg/ml)	37.91 (24.52, 44.11)	41.28 (30.73, 50.93)	0.515
25(OH)D (ug/ml)	13.72 (10.18, 23.33)	14.88 (13.8, 26.98)	0.515
Ca (mmol/L)	2.13 (2.10, 2.22)	2.14 (2.09, 2.19)	0.460
P (mmol/L)	1.01 (0.84, 1.12)	0.95 (0.93, 1.03)	0.633
Mg (mmol/L)	0.79 (0.76, 0.83)	0.82 (0.79, 0.84)	0.173
C-peptide (ng/ml)	1.17 (0.83, 1.54)	1.64 (1.03, 1.93)	0.068
Creatinine (umol/l)	66.5 (58.5, 69.75)	66 (61.25, 72.5)	0.897

*p < 0.05; Statistically significant *p* values are shown in bold; BMI: body mass index. Median (1st quartile, 3rd quartile) was used to show all data of both groups.

### Correlation Analysis

#### Correlations Between Femoral BMD and Serum Biochemical Makers, and Femoral Neck T Score

**FIGURE 2:**
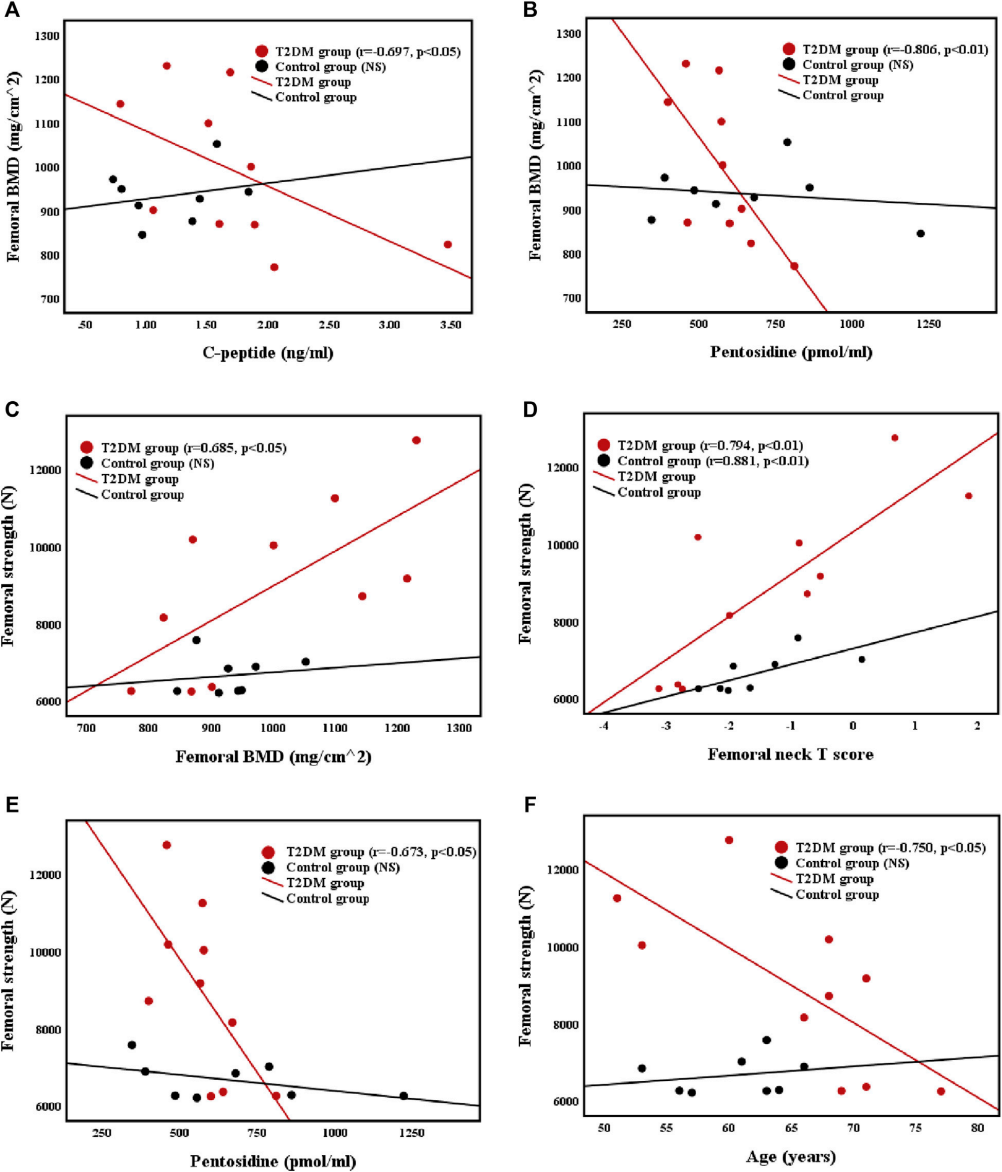
Correlations between **(A)** femoral BMD and C-peptide level **(B)** femoral BMD and pentosidine level **(C)** femoral strength and femoral BMD **(D)** femoral strength and femoral neck T score **(E)** femoral strength and pentosidine level, and **(F)** femoral strength and age in T2DM and control groups.

The relationships of the femoral BMD with C-peptide level and pentosidine level were illustrated in Figures 2A,B. The femoral BMD was negatively correlated with C-peptide (r = −0.697, *p* < 0.05) and pentosidine (r = −0.806, *p* < 0.01) levels and positively correlated with femoral T score (r = 0.818, *p* < 0.01) in T2DM group. However, the femoral BMD was not associated with C-peptide and pentosidine levels in control group. In addition, the femoral BMD was not related to other biochemical markers and basic body parameters in both groups.

#### Correlations Between Femoral Strength and Femoral BMD, Femoral Neck T Score, Pentosidine, and Age

The relationships of femoral strength with femoral BMD, femoral neck T score, pentosidine level, and age were presented in Figures 2C–F. Femoral strength was positively correlated with femoral BMD (r = 0.685, *p* < 0.05) and femoral neck T score (r = 0.794, *p* < 0.01), and negatively correlated with pentosidine level (r = −0.673, *p* < 0.05) and age (r = −0.750, *p* < 0.05) in T2DM group. Nevertheless, the femoral strength of control group was positively correlated with femoral neck T score (r = 0.881, *p* < 0.05), but was not related to femoral BMD or age. Furthermore, femoral strength was not related to other biochemical markers and basic body parameters in both groups.

#### Correlations Between Femoral Strength and BTMs

**FIGURE 3:**
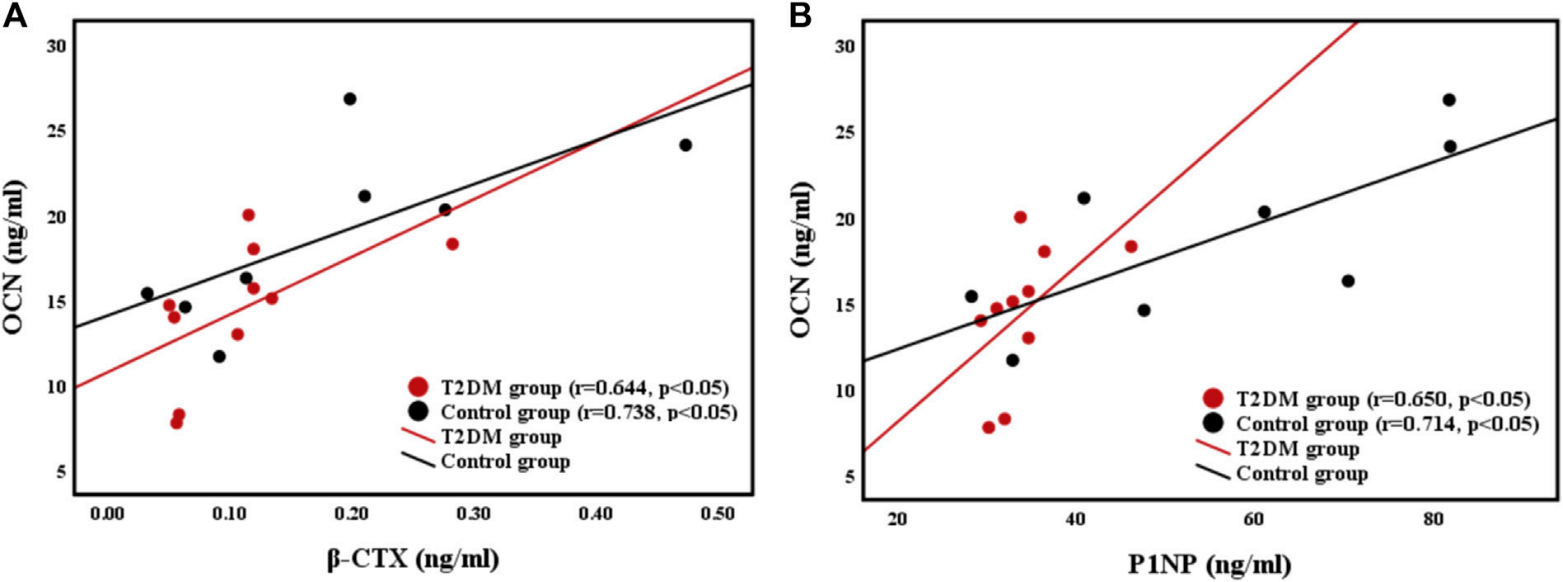
Relationships between **(A)** bone resorption marker β-CTX level and bone formation marker OCN level, and **(B)** bone formation maker P1NP level and OCN level.

The relationships of the OCN (bone formation marker) level with the β-CTX (bone resorption marker) level and P1NP level were shown in Figure 3. Femoral strength was not related to BTMs in both groups. However, the OCN level was positively correlated with the CTX level (r = 0.644, *p* < 0.05; r = 0.738, *p* < 0.05) and the P1NP level (r = 0.650, *p* < 0.05; r = 0.714, *p* < 0.05) in both groups.

### Multiple Linear Regression

Based on the analyses of difference and correlation, the parameters that were significantly related to femoral strength (*p* < 0.05) were selected as the independent variables of multiple linear regression. In addition, the parameters of significant differences (*p* < 0.05, HbA1c and P1NP) or potential significant differences (0.05 < *p* < 0.07, BMI and C-peptide) between T2DM and control groups were also involved, and femoral strength was the dependent variable of multiple linear regression. For the selection of parameters, although femoral BMD and femoral neck T score were both positively related to femoral strength, femoral neck T score was more related to femoral strength than femoral BMD. In order to avoid collinearity of the independent variables, only femoral neck T score was involved in the multiple linear regression.

Femoral neck T score was the common independent variable that was significantly related to femoral strength in both groups, thus it was used as the independent variable of the basic model. The selected independent variables were added into the models. Goodness-of-fit in the models was characterized by adjusted R-squared, as shown in Table 3.

**TABLE 3:** Multiple linear regression models of the T2DM and control groups.

Linear regression models	Adjusted R-squared	
	T2DM group	Control group
Femoral neck T score	0.632	0.661
Femoral neck T score + Pentosidine	**0.672**	0.618
Femoral neck T score + Pentosidine + Age	**0.733**	0.546
Femoral neck T score + Pentosidine + Age + HbA1c	0.896	0.397
Femoral neck T score + Pentosidine + Age + HbA1c + P1NP	**0.961**	**0.773**
Femoral neck T score + Pentosidine + Age + P1NP + HbA1c + BMI	0.958	**0.798**
Femoral neck T score + Pentosidine + Age + P1NP + HbA1c + BMI + C-peptide	0.942	—
Femoral neck T score + PINP	0.597	**0.888**
Femoral neck T score + P1NP + BMI	0.702	**0.930**

“-” implied that there were no outputs due to the problem of data structure. Independent variables with significant improvement of adjusted R-squared were marked in bold.

Comparison of the adjusted R-squared values of multiple linear regression models in Table 3 showed that the model (femoral neck T score + pentosidine + age + HbA1c + P1NP) of T2DM group (adjusted R-squared = 0.961) and model (femoral neck T score + P1NP + BMI) of control group (adjusted R-squared = 0.930) were the best models for predicting femoral strength. The best multiple linear regression models and their coefficients of the best models were shown in Table 4. Femoral neck T score and P1NP were the common predictors of femoral strength in two groups. Pentosidine, age and HbA1c played significant roles in predicting femoral strength of T2DM group, but BMI only presented a certain predictive effect on femoral strength of control group.

**TABLE 4:** The best models for predicting femoral strength of T2DM and control groups.

Models		Unstandardized coefficient B	Standardized coefficient β	Sig	95% confidential interval for B	
					Lower bound	Upper bound
T2DM group	Constant	29240.848	—	0.000	21403.464	37078.232
	Femoral neck T score	315.404	0.235	0.121	−131.132	761.940
	Pentosidine	−14.118	−0.754	0.003	−20.102	−8.135
	Age	−153.863	−0.572	0.007	−236.221	−71.505
	HbA1c	−879.085	−0.484	0.004	−1,279.626	−478.543
	P1NP	161.406	0.347	0.037	15.401	307.411
Control group	Constant	9,584.833	—	0.000	8,099.800	11069.867
	Femoral neck T score	745.051	1.083	0.001	528.312	961.790
	BMI	−40.206	−0.200	0.117	−96.163	15.750
	P1NP	−13.840	−0.508	0.011	−22.431	−5.250

## Discussion

Elderly male patients with T2DM and the age-matched control group were recruited in this study. Femoral strength was obtained *via* FEA method based on QCT images of the hip. Comprehensive biochemical measurements, including blood routine, trace elements, BTMs, and AGEs, were performed. Higher BMD (Nilsson et al., 2017), higher HbA1c (Karim et al., 2018), and lower bone turnover rate (Manavalan et al., 2012; Shu et al., 2012) in patients with T2DM were also confirmed in this study. Femoral strength in T2DM group was significantly higher than that in control group. Furthermore, multiple linear regression analysis was conducted to find out the best models for predicting femoral strength and the main influencing factors of femoral strength for T2DM and control groups.

BMD is regarded as one of the crucial factors in determining bone strength. No significant differences in femoral BMD and femoral neck T score were observed in T2DM and control groups. Some studies have indicated that BMI was higher in patients with T2DM than that in healthy subjects. High BMI was not completely beneficial to preventing bone loss. Although it played a role in protecting bone, the bone was not protected when BMI was in the overweight zone (BMI ≥25 kg/m^2^) (Cortet et al., 2019). Medians of BMI and BMD in T2DM group were high in our study, but the differences in BMD and BMI between the two groups were not statistically significant. Nevertheless, the femoral strength in T2DM group was significantly higher than that in control group, and femoral strength was positively correlated with the femoral BMD and femoral neck T score. Bone strength is mainly determined by the combination of BMD and bone quality. First, BMD was generally considered as the decisive factor of bone strength, as it could determine 70% of bone strength (National Institutes of Health Consensus Development Conference Statement, 2000). BMD of T2DM has always been controversial. In previous studies, reduced bone strength of T2DM was associated with low BMD (Tanaka et al., 2018; Hunt et al., 2019). T2DM group in the present study showed a higher BMD than control group. Therefore, high BMD causing high bone strength in T2DM group did not contradict with previous studies (Tanaka et al., 2018; Hunt et al., 2019), and it was highly reasonable. Second, bone quality is mainly determined by bone material properties and structure. In previous studies, low bone strength in T2DM patients derived from mechanical tests was associated with deterioration of bone microstructure (Karim et al., 2018; Hunt et al., 2019). T2DM could lead to deterioration of cancellous bone structure (Zeitoun et al., 2019). In this study, femoral strength based on QCT image and FEA simulation could not fully reflect bone microstructure characteristics, including decrease in trabecular thickness, increases in rod-trabecular and trabecular separation caused by T2DM (Karim et al., 2018; Hunt et al., 2019). It might be that the effects of impaired cancellous bone microstructure in T2DM group were weakened in the FEA simulation, so as to result in a high dependence of femoral strength on BMD. Therefore, the medians of BMD and femoral neck T score in patients with T2DM were higher than those of the control subjects, implying that femoral strength of T2DM patients is greater than that of individuals without T2DM.

In this study, the femoral BMD was negatively correlated with C-peptide in T2DM group. The absence of insulin leads to metabolic disorders and glucose metabolic barrier, and patients with T2DM presented a high glucose level. C-peptide was a product secreted by pancreatic β-cells, and it was used to represent insulin level (Li et al., 2013). In combination with the significantly high glucose and the negative correlation of glucose and C-peptide of T2DM group in this study, it was confirmed that the absence of insulin might lead to high glucose level. Similar to a previous finding (Li et al., 2013), our results showed that the femoral BMD was negatively correlated with C-peptide. This result indicated that the absence of C-peptide might lead to increase in femoral BMD. Although no significant difference was observed in C-peptide of both groups, the C-peptide level of T2DM group was lower than that of control group. Therefore, decreased C-peptide level might be a potential factor that helped increase femoral BMD and strength in T2DM group.

No differences in the total AGEs, pentosidine and sclerostin levels were observed between T2DM and control groups in the present study. Pentosidine was a typical AGEs, and serum pentosidine level was regarded as a valid marker of AGEs level because the formation of pentosidine required glycosylation and oxidation (Wang et al., 2002). Glycosylation could also affect bone strength (Saito et al., 2006). In our study, no significant difference in the serum pentosidine level was observed between both groups possibly because of metformin and insulin injection in patients with T2DM (Lapolla et al., 2005; Kanazawa et al., 2011). However, similar to a previous result (Wang, et al., 2002), the present study showed that the femoral strength and femoral BMD of T2DM group were both negatively correlated with pentosidine level. In addition, the pentosidine level was positively associated with the C-peptide level in T2DM group, and C-peptide level was used to represent insulin level (Li et al., 2013), thus decreased insulin secretion might lead to lower pentosidine level in T2DM group than that in control group. A previous study indicated that accumulation of AGEs in bone resulted in impairment of bone mineralization (Al-Khateeb et al., 2018). It might be the reason of low pentosidine level and high femoral BMD of T2DM group in the present study. In sum, our findings implied that low pentosidine level might be a cause of high femoral BMD in T2DM, consequently increasing femoral strength. Furthermore, although there was no significant difference in sclerostin level between two groups in the present study, the median of sclerostin level in control group was a little higher than that in T2DM group. A previous study had shown that the sclerostin level in diabetic rats decreased with aging and the deterioration of glycemic control, which indicated that sclerostin level might be related to the glycemic control and age of rats (Pereira et al., 2017). Therefore, a higher sclerostin level of control group than T2DM group in the present study might be related to the stable glycemic control and younger age.

Aging was considered an important factor of bone loss and decreased bone strength (Lang et al., 2012). The present study showed that age was negatively correlated with femoral strength in T2DM group. This result implied that the bone strength of T2DM might decline with aging and it agreed with the finding that both men and women lose their bone strength with aging (Lang et al., 2012). However, age had no significant correlations with the femoral BMD and femoral strength in control group. A previous research revealed that DM could change bone microstructure and bone spatial distribution (Burghardt et al., 2010), which affected bone mechanical properties. Therefore, the correlation between age and femoral strength in T2DM group might be due to the combined contribution of age and bone structure. The specific mechanism of the combined contribution should be confirmed in further studies.

A previous study reported that increasing BTMs in old people could lead to exacerbation of hip bone loss (Bauer et al., 2009). In the present study, the P1NP level in T2DM group was strongly lower than that in control group, and the medians of β-CTX and OCN levels in T2DM group were lower than those in control group, but they did not have statistical significance. Moreover, bone resorption and formation markers were positively correlated in both groups, as shown in Figure 3. Similar to previous results (Shu et al., 2012; Farr et al., 2014), our findings indicated that the bone turnover rate in T2DM group was lower than that in control group, and Figure 3 showed that bone turnover was balanced in both groups. Uncoupled or unbalanced bone turnover could cause severe changes in bone mass (Stein et al., 2010), thus balanced bone turnover might prevent loss of bone mass. The bone turnover rate in T2DM group was lower than that in control group possibly because high glucose level suppresses the functions of osteoclasts and osteoblasts (Wittrant et al., 2008). Although low bone turnover rate could reduce the bone loss rate, it also increased bone fragility because of the irreparable accumulation of microdamage. Nevertheless, a previous study suggested that rapid bone turnover might cause younger and less dense mineralized bone to replace more dense mineralized bone; thus, bone stiffness and strength could be reduced (Seeman and Delmas, 2006). In addition, OCN and β-CTX had a positive correlation in both groups, implying that bone turnover was balanced in both groups. In summary, bone turnover was balanced in both groups, and the femoral BMD and strength of T2DM group were higher than those of control group likely because of a low bone turnover rate.

Multiple linear regression analysis showed that the best predictive models of T2DM and control groups could accurately predict the femoral strength. Femoral neck T score and P1NP were the common predictors of femoral strength in both groups, which indicated that BMD and bone formation level played important roles in predicting femoral strength. Furthermore, pentosidine, age and HbA1c presented significant effects on predicting femoral strength in T2DM group, which implied that AGEs, age and blood glucose might be the important influencing factors on bone strength of T2DM. However, blood glucose and AGEs were not significantly related to the predication of femoral strength, which might be caused by the normal blood glucose level in control group due to increase in AGEs level with high blood glucose level (Farlay et al., 2016). Age was only associated with the prediction of bone strength in T2DM group, which suggested that age played a more significant role in the change of bone mass in T2DM patients than that in controls. In addition, BMI only showed a certain predictive potential in control group. Higher BMI could decrease bone loss (Cortet et al., 2019), thus BMI as a predictor of femoral strength of control group might be reasonable in the present study. For T2DM group, high glucose could cause some complicated situation of bone tissue, such as metabolic disorders and the accumulation of pentosidine, which might weaken the effect of BMI on femoral strength. Above results indicated that the predictors of femoral strength for T2DM patients and non-T2DM people were different. Moreover, in all the predictors of the best model for the controls, femoral neck T score presented the strongest predictive ability, which was consistent with a previous study (Keaveny et al., 2010). However, the ability of femoral neck T score for predicting femoral strength was weaker than other predictors of the best model for T2DM group. Multiple regression differed from correlation analysis, and regression model was a result of the interaction and control of independent variables (Gogtay et al., 2017). Thus, other predictors in the predictive model of femoral strength for T2DM could weaken the significance of femoral neck T score in multiple linear regression so as to obtain the optimized predictive model. In sum, multiple linear regression analysis stated that femoral neck T score contributed to predicting femoral strength, and P1NP, pentosidine, age and HbA1c also played important roles in evaluating femoral strength in T2DM. Therefore, in clinical assessment of bone fracture risk of T2DM, besides femoral neck T score, more attention should be paid on P1NP, pentosidine, age and HbA1c.

To date, many studies have indicated that T2DM could lead to increase of bone fragility (Napoli et al., 2017; Acevedo et al., 2018). Although there was a higher femoral strength in T2DM group than control group in the present study, it did not contradict with the high bone fracture risk in T2DM. Increase in bone fragility of T2DM might be one cause of high bone fracture risk. Bone fragility could be defined by biomechanical parameters, including ultimate force, ultimate displacement and work to failure (energy absorption or toughness) (Turner, 2002). Bone elasticity and plasticity both contributed to fracture (Seeman, 2008). Toughness derived from bone plasticity could compensate bone fragility. A previous study showed that accumulation of AGEs in T2DM bone could reduce bone plasticity and toughness, which led to decrease in bone ductility and increase in bone fragility (Tang and Vashishth, 2011). Furthermore, bone strength and stiffness increased with the increase in BMD, but bone toughness decreased with the increase in BMD (Seeman and Delmas, 2006). It implied that bone strength and fragility could both increase with the increase of BMD. Therefore, when bone strength is high, increased bone fracture risk may result from the significant decrease in bone plasticity and increase in bone fragility. In addition, there were many factors that may be related to the high bone fracture risk of T2DM (Schwartz and Sellmeyer, 2007; Isidro and Ruano, 2010; Moseley, 2012). Compared to the non-diabetes, the fall risk of T2DM patients was higher. Moreover, the complications of T2DM could increase the fall risk of patients, such as syncope directly caused by hypoglycemia, falls caused by autonomic neuropathy, orthostatic hypotension, peripheral neuropathy, and retinopathy, thus increase in fall risk might be an important factor of high bone fracture risk of T2DM (Schwartz et al., 2008; Moseley, 2012). Taken together, increase in bone fracture risk of T2DM is not only related to the intrinsic mechanical properties of bone, but also associated with the high fall risk of T2DM patients. Furthermore, high bone strength and increased bone fragility might both exist at the same time in T2DM bone.

This study has some limitations. First, the number of subjects was less than our expectation because of the strict recruitment criteria and the absence of several volunteers in some processes. Small sample size might lead to undervalued differences in data between two groups. In addition, this study was unable to assess the roles of drug therapies in predicting femoral strength due to the small sample size and the different drugs taken by T2DM patients. It was expected that future studies with a large sample size study could overcome the difficulties faced by investigations of drug therapies for T2DM patients, so as to conduct a detailed and comprehensive analysis on the effects of different drug therapies and treatment duration on the prediction of bone strength in T2DM. Second, bone microstructure played a certain role in predicting bone strength. Although our study found out the significant influencing factors of femoral strength, it lacked femoral microstructure analysis due to the limitation of clinical CT scanner for imaging femoral microstructure. Investigation of femoral microstructure *via* high resolution magnetic resonance imaging in patients with T2DM might be valuable for evaluating femoral strength (Chang et al., 2017). Third, the results of the present study suggested that AGEs, blood glucose and age were all the important predictors of femoral strength in T2DM, but the detailed and in-depth mechanism research of predicting femoral strength was absent in this study. Therefore, further molecular and compositional investigations in femurs might contribute to the understanding of deep predictive mechanism of the predictors.

In conclusion, femoral strength was significantly higher in elderly men with T2DM than those without T2DM (range of age: 51–77 years), and this finding might be related to higher BMD and lower bone turnover rate in T2DM. C-peptide, pentosidine, and age had harmful effects on the femoral BMD and strength of patients with T2DM. Furthermore, besides femoral neck T score and P1NP, pentosidine, age and HbA1c all played significant roles in predicting femoral strength in T2DM. This study provided important references for predicting femoral strength and evaluating bone fracture risk in clinics.

## Data Availability

The raw data supporting the conclusion of this article will be made available by the authors, without undue reservation.
